# Older adults’ community participation, physical activity, and social interactions during and following COVID-19 restrictions in Australia: a mixed methods approach

**DOI:** 10.1186/s12889-023-15093-0

**Published:** 2023-01-25

**Authors:** Claire Gough, Chris Barr, Lucy K Lewis, Claire Hutchinson, Anthony Maeder, Stacey George

**Affiliations:** 1 College of Nursing and Health Sciences, Flinders University, Adelaide, Australia; 2 Flinders Digital Health Research Centre, Flinders University, Adelaide, Australia; 3 Caring Futures Institute, Flinders University, Adelaide, Australia

**Keywords:** Community participation, Older adults, Physical activity, Social interactions, COVID-19

## Abstract

**Background:**

With the increasing age of the global population, key components of healthy ageing including community, physical, and social participation continue to gain traction. However, management of the COVID-19 pandemic aimed to protect older adults and reduce the spread of the virus, this restricted community participation and reduced the opportunities for social interaction.

**Methods:**

This mixed methods study investigates community dwelling older adults’ community participation; physical activity and social interaction prior to, during, and following the COVID-19 lockdown in Adelaide, Australia. Twenty-six community dwelling older adults were monitored over three time-points between November 2018 and October 2020, with Global Positioning Systems, accelerometry and self-reported diaries. In addition, nineteen participants completed semi-structured interviews.

**Results:**

Community participation varied across the three time points, with significant reduction in the number of trips taken out-of-home (p = 0.021), social interactions (p = 0.001) and sleep quality (p = 0.008) during restrictions. Five themes were identified to explain personal experiences of community participation during restrictions: *(1) Reframing of meaning, (2) Redefining to maintain activities, (3) Revision of risk, (4) Reflection and renewal* and *(5) Future planning.*

**Conclusion:**

During COVID-19 the physical and social activities of community dwelling older adults changed. Services that support older adults to adapt their activities   , considering their capacities and preferences, to facilitate community participation are required.

**Supplementary Information:**

The online version contains supplementary material available at 10.1186/s12889-023-15093-0.

## Background

With the global ageing population, care for older people has become a focal point for governments, researchers and healthcare services [1]. In 2019, internationally there were 703 million people (nine per-cent of the population) aged over 65 years, with this number expected to reach 1.5 billion by 2050 [2]. In 2017, approximately 3.8 million Australian’s were aged 65 years and over (16%) [3], corresponding health costs of ageing are projected to reach $35.3 billion (AUD) by 2051 [4]. Healthy ageing, and promotion of ageing in place is becoming increasingly important. In conjunction, the importance of key components of healthy ageing for older adults such as community, physical, and social participation are gaining traction. Community participation has been described as engagement in activities outside of the home which are social, complex in nature and non-domestic [5]. Social participation describes activities that provide interaction with other people in a society or community [6]. Older adults who do not participate in community or social activities are at higher risk of functional disability, lower health related quality of life (HRQOL), increased use of healthcare services, loneliness, social isolation, and depression [7, 8].

The management of the COVID-19 global pandemic and measures implemented to reduce the spread of the virus has restricted community participation for older adults [9]. It was evident early in the pandemic that older adults were at a higher risk of morbidity and mortality from COVID-19 [10]. To prevent healthcare services from becoming overwhelmed, Australia, like many other countries introduced social distancing restrictions [11, 12]. While these restrictions were somewhat successful in curbing the spread of the virus, they presented further challenges for older adults who are already at high risk of loneliness and social isolation [13, 14]. Prior to the global pandemic, 19% of Australian adults aged 75 years and over reported feeling lonely and socially isolated (8%) [15]. Given the high prevalence of loneliness and social isolation prior to the pandemic, it was important to explore how social distancing restrictions may have impacted the behaviours and subsequent wellbeing of older adults. The use of mixed methods to combine quantitative findings with the perspectives of older adults could provide new insight into the behaviours and coping mechanisms during this challenging and unprecedented time.

A vast amount of research has been published detailing the impacts of COVID-19 worldwide. Following the declaration of a global pandemic by the World Health Organisation (WHO), physical activity (PA) including ‘any bodily movement produced by skeletal muscles that required energy expenditure’ [16] and global step counts reduced [17, 18]. Which corresponded with increased sedentary time, declining mental health, loneliness, and poor sleep quality [19, 20]. These studies provide useful insight into measures of health, however, due to the large sample sizes, they used questionnaires to measure previous physical activities which may be subject to recall bias [21]. Research that uses a combination of real-time measures (i.e., Global Positioning Systems (GPS) and accelerometry) and qualitative measures can provide a more holistic picture of community participation [22].

Therefore, the aims of this mixed methods study were to:

1) investigate community participation; PA and social interaction for a cohort of older adults living in the community, prior to, during, and post lockdown (during social distancing restrictions),

2) understand the facilitators and barriers to physical and social community participation during restrictions from participants’ perspective, and

3) examine the associations between restrictions and health-related factors including quality of life, sleep quality, and loneliness.

## Methods

This study used a convergent mixed methods approach [23], quantitative data from observations and qualitative data from semi-structured interviews were collected, analysed separately and compared. The mixed methods approach allowed for the investigation of complex processes that contribute to community participation in relation to health, providing a holistic overview [24]. Ethical approval was gained from the Flinders University Social and Behavioural Research Ethics committee (protocol no. 8176). An amendment was approved to return to previous participants who had completed a community participation baseline study [22] and to deliver devices and resources to participant’s letterboxes to maintain social distancing.

### COVID-19 australian context

**Fig. 1:**
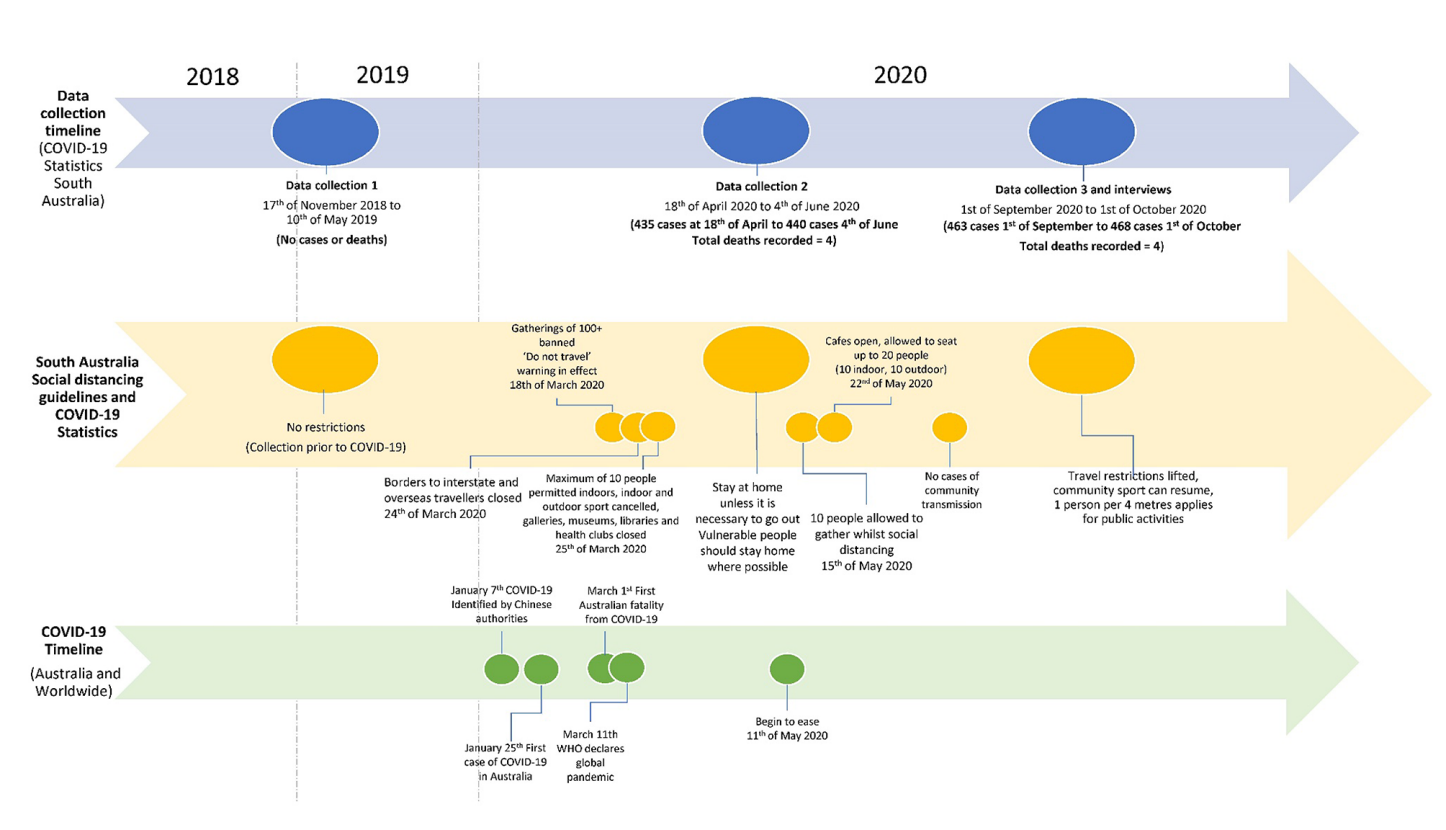
Timeline of data collections, restrictions, and COVID-19 statistics in Australia and worldwide

Australia’s experience of the global COVID-19 pandemic, lockdowns, and social distancing restrictions varied from that of other countries, likewise the experience between states across Australia was not homogenous. To provide context, a timeline is provided in Fig. 1 detailing the times of data collection and the corresponding social distancing restrictions in place.

### Participants

Participants were required to be aged 65 years or older, live-in metropolitan Adelaide, Australia, be able to walk independently with or without walking aids, speak and understand English and have sufficient cognition to understand study instructions. Individuals living in residential care facilities were excluded due to restrictions on community participation. Participants were recruited using flyers through local councils, community centres, organisations for older adults and via social media. Potential participants were invited to contact the Principal Investigator who screened potential eligibility over the phone. Cognitive capacity was assessed using the Standardised Mini Mental State Examination (SMMSE) prior to obtaining consent, with scores of 25 and over required to participate [25].

### Outcomes

The outcomes and related measures for this study have been previously published in detail elsewhere [22], however, a contextual overview is provided below.

#### Community participation

Community participation was measured using GPS (Qstarz BT1000XT) to calculate the number of trips away from home, type of location visited and the number of in- and out-of-home activities over three separate 7-day monitoring periods. The locations visited were identified and viewed on the street view of Google maps [26]. The types of location were grouped into residential, recreational, commercial, health, local walk/greenspace, Central Business District (CBD) and place of worship [22, 27]. Locational data were cross referenced with self-reported activity diaries to determine the purpose of the visit and to identify social interactions.

To provide context of community participation, self-reported participation diaries were completed by participants. These diaries reported the date, time, activity, duration, location, and social interactions that occurred outside of the home, as well as times of sleep and non-wear, an excerpt is provided in Appendix 1. This information was cross-checked against the quantitative data to identify activities and ensure accuracy of location where GPS data were missing.

#### Physical activity

Physical activity was measured using wrist-worn GeneActiv triaxial accelerometers, (24 h, 7-day protocol). These devices have been deemed reliable and valid for classifying the intensity of PA in adults [28]. Raw GeneActiv. Bin files were converted into 60-second epoch files and analysed using Cobra software to determine daily PA (Francois Frayasse, University of South Australia). Cut points were used (adjusted for the sampling frequency) to identify times spent in light, moderate or vigorous PA (light 283, moderate 605, vigorous 1697) [28]. Sleep was identified using self-reported diaries and visual analysis of the activity trace and subsequently excluded from analysis.

#### Social interactions

The number of social interactions were self-reported in activity diaries by participants, allowing for the total number and location of interactions to be identified across the three monitoring periods.

#### Health-related factors

The Assessment of Quality of Life-8 Dimension scale (AQOL-8D) [29] was used to measure HRQOL and the associated dimensions. Sleep quality was measured using the Pittsburgh Sleep Quality Index (PSQI) [30] and levels of loneliness were measured using the de Jong Gierveld loneliness scale [31].

#### Facilitators and barriers

Participants were invited to take part in a semi-structured interview to provide their experiences of community participation prior to and during times of lockdown and social distancing. Interviews were carried out either in person (as social distancing restrictions allowed) or over the phone. Questions included, *‘can you describe ways you did things differently to keep active physically and socially during times of social distancing restrictions?’* A detailed interview guide can be found in Appendix 2.

## Procedure

The procedure, data processing and analysis have been previously published [22]. These procedures were repeated over three separate 7-day monitoring periods as detailed in Fig. 1 and data compared between time-points. Demographic questionnaires were completed at baseline prior to data collection.

## Data analysis

To be included in analysis, GPS data required a minimum of eight hours per day, complete for five of the seven days monitored, with accelerometer data required for at least eight hours of waking time for four days inclusive of one weekend day [32–34]. Individual participant data was matched across the three time-points, and SPSS software was used for the analysis [35]. All data were checked for normality using Z scores. Descriptive statistics were generated for all variables (means and SD for normally distributed data, median and inter-quartile range (IQR) for skewed data). Mann Whitney U tests were completed to compare baseline with the two subsequent time points. Non-parametric Friedman ANOVA tests were performed to detect differences between paired baseline, lockdown, and post lockdown measures with post-hoc analyses used to determine significance. Alpha was set at < 0.05 [36].

Qualitative data were used to determine the validity of the quantitative results against lived experience [24]. An integrated parallel approach to data collection was taken, whereby quantitative and qualitative data were collected at similar times, and data analysed after the completion of data collection [24]. The two data sets were analysed separately (Phase 1) and merged to determine similarities and differences (Phase 2) [23].

### Phase 1

Interviews were recorded and transcribed verbatim. Transcripts were imported into NVIVO 12 Pro for content analysis [37] which was driven by study research aims. Template analysis [38, 39] was carried out by the Principal Investigator (CG) and Senior Author (SG) who read through a subsample of transcripts and identified an initial list of themes. An interpretive phenomenological approach was used to discover the meaning of the lived experience [40, 41]. This approach is useful in areas where not much is known and has been used successfully in disability and rehabilitation research [42, 43]. For this study, the experience of social distancing and social isolation was investigated using semi-structured interviews, consistent with the interpretative phenomenological approach [40] and appropriate for generating rich descriptions.

### Phase 2

After identifying themes from the interview data, a concurrent mixed methodology approach was used. Quantitative and qualitative data, including GPS locational data, minutes of physical activity, and self-reported activity diaries, were integrated to compare and combine the experiences of community participation for older adults. Aligned with Creswell (2018), mixed methods are useful where one data source could be insufficient to explore the complexity of a theme. An advantage of mixed methods is that the combination of methods can offset the inherent weaknesses of both quantitative and qualitative approaches, such as loss of GPS data and memory recall bias for subjective measures [44]. This approach allowed for the inclusion of the voices of the participants which was integral to understanding their experiences and reasons for behaviour change.

## Results

### Participant characteristics

A subsample of 27 participants from the original sample (n = 44) who completed the baseline study [22] participated in all three data collection time points. One participant’s data were excluded from analysis as the GPS data did not meet quality standards. Therefore, data from 26 participants with complete quantitative data and 19 participants who completed semi-structured interviews are included in the results. On average participants were aged 75 years (SD 5.2), were predominantly female (n = 21, 81%), and lived alone (n = 15, 58%). All participants demonstrated normal cognition with an average SMMSE score of 28.9 (SD 1.5). Participants reported two chronic conditions on average with 12% reporting excellent health (n = 3). Full details of participant characteristics can be found in Table 1.

**Table 1:** Participant characteristics

Characteristic	All participants (n = 26)	Interview participants (n = 19)
Gender (M:F) n (%)	5: 21 (19: 81)	3:16 (16:84)
Age mean (SD) years	75.0 (5.2)	74.0 (5)
BMI mean (SD) Underweight n (%) Normal n (%) Overweight n (%) Obese n (%)	28.3 (4.4) 0.0 (0) 8.0 (30.8) 7.0 (26.9) 11.0 (42.3)	30.0 (5) 0.0 (0) 5.0 (26.3) 3.0 (15.8) 11.0 (57.9)
BMI mean (SD)	28.3 (4.4)	30.0 (5)
Underweight n (%)	0.0 (0)	0.0 (0)
Normal n (%)	8.0 (30.8)	5.0 (26.3)
Overweight n (%)	7.0 (26.9)	3.0 (15.8)
Obese n (%)	11.0 (42.3)	11.0 (57.9)
Marital status n (%)		
Single/never married	1.0 (3.8)	1.0 (5.3)
Separated/divorced	9.0 (34.6)	8.0 (42.1)
Widowed	8.0 (30.8)	7.0 (36.8)
Married/defacto	8.0 (30.8)	3.0 (15.8)
Education level n (%)		
High-school	5.0 (19.2)	4.0 (21.1)
Post-secondary	10.0 (38.5)	6.0 (31.6)
Bachelor degree	7.0 (26.9)	3.0 (15.8)
Post-graduate	4.0 (15.4)	6.0 (31.6)
Employment status n (%)		
Employed	2.0 (7.6)	1.0 (5.3)
Retired	24.0 (92.3)	18.0 (94.7)
Volunteer n (%)	15.0 (57.7)	14.0 (74)
No. of co-morbidities mean (SD)	2.0 (1.4)	2.0 (1.6)
Self-rated general health n (%)		
Excellent	3.0 (11.5)	2.0 (10.5)
Very good	3.0 (11.5)	2.0 (10.5)
Good	7.0 (26.9)	6.0 (31.6)
Fair	0.0 (0)	0.0 (0)
Poor	0.0 (0)	0.0 (0)
SMMSE mean (SD)	28.9 (1.5)	29.0 (1.6)

(Standard deviation (SD), Body mass index (BMI), Defacto: a relationship where two people are not married and live together as a couple on a genuine domestic basis, Standardised Mini Mental State Examination (SMMSE))

### Community participation

During the lockdown period participants took less trips out of home, compared with baseline, and the number of in-home activities increased (Table 2).

**Table 2:** Community participation trips out of home and activities at baseline, during and post lockdown

Variable	Baseline	Lockdown	Post lockdown
Trips out of home (n/week)*	15 (9–19)	11.5 (7–16)	13 (9–17)
Participants reporting not leaving the house for a whole day (total)	9	14	10
Number of days participants did not leave the house	16	26	15
In-home activities (n/week)*	12.5 (4–17)	36.5 (20–51)	31 (15–47)
Out-of-home activities (n/week)*	18.5 (13–25)	13.5 (8–20)	20 (12–21)
Total activities reported (n/week)*	33.5 (24–37)	47 (34–67)	49.5 (31–61)

(* Median (IQR))

The most frequently visited locations at baseline were commercial (median (IQR)) 5.5 (3–8) followed by recreational 3 (2–7), residential 2 (0–3), local walk greenspace 2 (0–8), CBD 1 (0–3), with no visits to places of worship identified. During COVID-19 lockdown the most visited location type was commercial 6 (2–7), followed by local walk/greenspace 4 (1–10), residential 1 (0–3) with no visits to recreational, health, CBD, or places of worship identified. Post lockdown the most visited location was commercial 7 (3–10), followed by local walk greenspace 3 (0–6), recreational 3 (1–4), residential 1 (0–2) and health 1 (0–2) with no visits to CBD or places of worship detected. There was a significant difference in the number of trips taken out-of-home between monitoring periods (p = 0.012). Significant post-hoc pairwise calculations identified a reduction in the number of trips taken out-of-home from baseline to during lockdown (p = 0.021).

### Physical activity

Total minutes of MVPA reduced from baseline to lockdown for 73% (n = 19) participants, with a decrease in daily MVPA from post lockdown to lockdown for 54% of the participants (n = 14) as reported in Table 3. There was a significant difference in the daily MVPA across the three time-points (p = 0.030), but when adjusted for multiple comparisons the post hoc pairwise comparisons found no significant differences (baseline to post lockdown p = 0.055, baseline to lockdown p = 0.080).

**Table 3:** Participant activity behaviours at baseline, during lockdown and post lockdown (min/d)

Activity behaviour (min/day)	Baseline	Lockdown	Post lockdown
Sedentary time (mean (SD))	652 (99)	685 (87)	680 (92)
Light PA*	252 (181–300)	205 (163–265)	210 (155–272)
Moderate PA*	69 (33–108)	57 (34–87)	55 (26–84)
Vigorous PA*	0 (0-1.5)	0 (0–1)	0 (0)
MVPA*	69 (33–110)	57 (35–87)	55 (26–84)

(MVPA Moderate to Vigorous physical activity; PA Physical activity; *Median (IQR); +Mean (SD))

### Social interactions

Self-reported social interactions median (IQR) was 15.5 (8–20) at baseline, 9 (5–13) during lockdown and 11 (7–13) post lockdown. The locations of social interactions are provided in Table 4. Friedman ANOVA post-hoc pairwise calculations identified a significant reduction in the total number of social interactions reported at baseline and during lockdown (p = 0.001).

**Table 4:** Locations of participant social interaction at baseline, during and post lockdown

Social interaction location*	Baseline	Lockdown	Post lockdown
Home (n/week)	1.5 (0–5)	2.5 (1–5)	0 (0–2)
Residential (n/week)	2 (0–4)	1 (0–2)	1 (0–2)
Recreational (n/week)	2 (1–5)	0 (0–1)	2 (0–3)
Commercial (n/week)	3 (1–4)	1 (0–2)	2 (2–4)
Health (n/week)	0 (0–1)	0 (0)	1 (0–2)
Local walk/greenspace (n/week)	0 (0–2)	1 (0–3)	0.5 (0-1.5)
CBD (n/week)	1 (0–2)	0 (0)	0 (0–1)
Place of worship (n/week)	0 (0–1)	0 (0)	0 (0)

(*Median (IQR))

### Health-related factors

There was no significant difference in participant HRQOL across the three time-points (Table 5). There was a significant reduction in sleep quality from baseline (5.0 SD 3.0) to lockdown (6.0 SD 3.0) (p = 0.005) during lockdown and an insignificant increase from lockdown to post-lockdown (5 SD 3) (p = 0.677). There were no significant differences in loneliness between baseline (1.5 SD 1.4), during (1.8 SD 1.2) and post lockdown (1.8 SD 1.6).

**Table 5:** Dimensions of HRQOL at baseline, during social distancing and following the easing of restrictions

Mean (SD)	Baseline	Lockdown	Post lockdown
HRQOL total	85 (7)	83 (8)	82 (9)
HRQOL dimensions			
Independent living	91 (10)	90 (10)	89 (10)
Happiness	81 (13)	80 (10)	79 (10)
Mental health	81 (9)	81 (7)	81 (7)
Coping	80 (12)	79 (10)	77 (10)
Relationships	89 (7)	85 (10)	86 (10)
Self-worth	87 (11)	89 (10)	78 (52)
Pain	82 (24)	81 (26)	77 (24)
Senses	86 (8)	84 (11)	83 (10)
PSD score	87 (10	86 (11)	84 (10
MSD score	84 (8)	83 (7)	81 (11)

(Super dimensions; Physical super dimension (PSD) inclusive of independent living, pain, and senses variables. Psycho-social super dimension (MSD) inclusive of mental health, happiness, coping, relationships and self-worth variables [45]).

### Perceptions of community participation

**Fig. 2:**
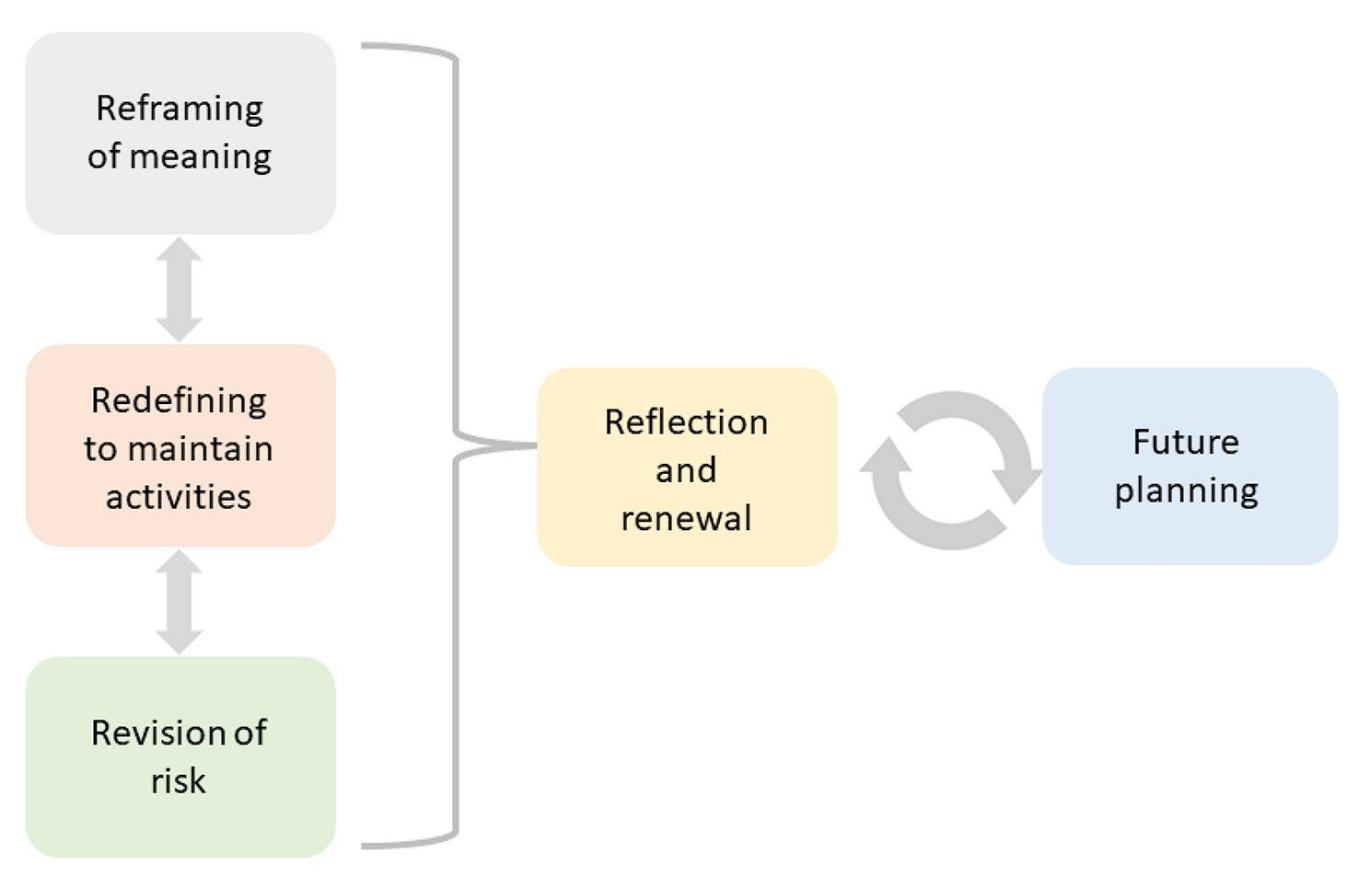
Interaction of qualitative themes

Qualitative data were collected until saturation was reached (n = 19), with five main themes emerging from the transcripts. These themes described participants’ perspectives of their community participation and identified differences in experiences between participants. The themes were *‘Reframing of meaning’, ‘Redefining to maintain activities’, ‘Revision of risk’, ‘Reflection and renewal’* and ‘*Future planning’*. The interaction between these themes, and the importance of reflection and renewal in the ongoing process of redefining community participation and future planning is illustrated in Fig. 2.

## Reframing of meaning

‘Reframing of meaning’ refers to changes in the meaning of activities, their setup, environment, and their importance in the lives of participants. Meanings of activities were framed in both positive and negative ways, with some acknowledging how different their social interactions were in the context of social distancing restrictions:

*I did see people, but it’s not the way I would normally socialise because there were no lunches with mates, and no work/volunteering-related stuff* (Participant 3, 69-year-old female living alone).

Whilst others reported the activities they had been participating in prior to restrictions perhaps weren’t meaningful and fulfilling to them, restrictions provided the opportunity to complete other activities:

*I found the whole thing easy because I got jobs done… yeah, jobs that I had been waiting to do for so long, so I was grateful for the lockdown* (Participant 7, 73-year-old female living alone).

Several participants reported that one of the hardest things was not being able to go out and get a *‘good coffee’* whilst sitting down with a friend and having a chat. The process of getting a coffee became an organised, regimented activity, inclusive of incidental interactions with others, with one participant describing this made them feel special:

*So, every day, I would bop down and get a proper coffee, didn’t touch anything, paid in advance so that I got Rolls-Royce treatment because I was the one that had paid– they all knew my name and they made nice coffee for me, and that was a real motivator because I could say g’day to people on the way down* (Participant 31, 71-year-old female living alone).

The importance of activities such as getting a coffee, acted as a motivator for individuals and made them realise, and appreciate the incidental social interactions they experienced when leaving the house.

Following the easing of restrictions, some participants reframed the meaning of their activities in terms of what it was they liked about attending. For some, this highlighted that it was incidental chats in communal spaces that motivated them to attend:

*I really don’t like not being able to make a coffee – have a communal kitchen and do the communal things together. I realise that a large part of that activity was being communal, so we’d go into the kitchen and have a chat while waiting for the kettle to boil or the microwave, and they’re not allowed* (Participant 33, 71-year-old female living alone).

The process of reframing made participants consider the value of their activities, change the way they participated and, in some cases, stop attending altogether.

## Redefining to maintain activities

Redefining to maintain physical and social activity (detailed below) describes the conscious adaptations participants made to continue activities during lockdown when recreational facilities were closed. Redefining activities to ‘keep them’ was reported by 17 participants, and for some the ability to ‘redefine’ these activities was a coping mechanism closely related to feelings of control.

### Redefining physical activity

With gyms closed and organised exercise classes cancelled some participants simply replaced their physical activities:

*Instead of going to work related things, I found myself going for more walks because I just needed to get out of the house* (Participant 3, 69-year-old female living alone).

Many participants reported leaving the house with the intent to walk purely for exercise to replace previous commitments:

*I did go out walking, as the exercises classes I went to stopped, so I was walking about three times a week* (Participant 14, 72-year-old female living alone).

Yet some identified that the intensity was *‘not the same,’* (Participant 17, 68-year-old female living with son) and that exercising alone was not as enjoyable as attending a group. Many participants looked to technology to redefine their PA, using the internet to find resources:

*Initially I was doing a lot of exercises from YouTube on the screen, as well as walking and that was great, so I got my steps and I got my exercise* (Participant 19, 72-year-old female living with son).

In contrast there was a portion of the cohort who made no attempt to redefine PA in their routine during this time:

*I was very bad. I sat on my bum and didn’t do enough, but I’ve now got a large knee brace on my right knee because various things that were going wrong with me… I didn’t exercise enough, and I don’t like exercise, I exercise because I have to, not because I want to and I’m afraid I did less* (Participant 33, 71-year-old female living alone).

### Redefining social activity

Participants were aware of the importance of social interactions and were conscious to retain them. Social interaction was maintained by increasing the effort of reaching out to others, and often re-defined using technologies that allowed for virtual face-to-face interactions:

*Initially I made a point of ringing two or three people every day just so I could get some human interaction… I very quickly learned how to use Zoom and talk to my daughter online and do things with my grandchildren and that kind of thing* (Participant 19, 72-year-old female living with son).

The inclusion of social media and technologies were enjoyable for some participants during this time:

*We were doing lots of Facetiming. And we were having the loveliest time. Especially doing those little icons where you could stick funny faces on and things. It was good because we sort of kept in touch* (Participant 20, 71-year-old female living with friend).

The environment and social activities were redefined regularly in the community:

*I would talk to the neighbours, and I started doing a lot of cooking and baking. The girl in that unit is still in the workforce, she was working from home. So, I’d make something and go around and give them all something. Then she’d make something. One day, she knocked at the door and she said, “Grab a cup of coffee and a chair. We’re going to sit out on the driveway.” So, the three of us did that, and that was nice* (Participant 14, 71-year-old female living alone).

Participants reported taking part in activities they wouldn’t have previously such as having a *‘glass of wine together on the street in front of our* houses’ (Participant 31, 71-year-old female living alone). Despite the venues, length of time, and social activities changing over this period, social interactions were prioritised. Participants planned the modifications of their activities to maintain them whilst following social distancing guidelines. However, some participants also felt that restrictions were open to interpretation and activities could be maintained if appropriate social distancing was performed:

*I missed being able to go to the coffee shop, and things like this, but we found ways around that a little bit. You know, picking up a coffee, and we went and sat up at the memorial park there apart from one another, and stuff like that. That was quite enjoyable, to do that. Before, it was like, take something with you, if we wanted to go and have a catch-up, I’d cut sandwiches and we’d take a couple of chairs and sit down along the beachfront. And I found that you’re a little bit up in the air to whether that was quite allowable, but I had police go past, and they’d just smile. But there was just two of us sitting there on a chair, good distance apart.* (Participant 34, 77-year-old female living alone).

It was apparent that participants who didn’t redefine physical or social activities experienced social isolation and feelings of loneliness:

*I did feel isolation and lonely at times… I would wake up about 5:00 o’clock in the morning. And suddenly you think about everything that you shouldn’t be thinking about. And it always seemed to be magnified, because you think well, I don’t know who I can talk to. I can’t go out and have a cup of coffee with anyone. So, I did find that a bit – I mean I didn’t get the tearies. But I did find it was a negative. Yes, it was a negative. And then I found I couldn’t be bothered doing the things that I wanted to do* (Participant 20, 71-year-old female living alone).

### Combining physical and social activity

Participants described making a conscious effort to combine their physical and social activities to maintain them. These combined activities often occurred outside to follow social distancing guidelines and were enjoyed by participants:

*Thank goodness I have a couple of friends who like walking, so I walked a lot in company; so we were arm’s length apart, we can still talk, but we could go for long walks and just get out in the fresh air and I’d go on my own sometimes and I’d find other people – just seeing someone else out on the street doing what you’re doing and they’re waving and saying hello even if you don’t know them, made me feel better* (Participant 19, 72-year-old female living alone).

## Revision of risk

‘Revision of risk’ describes the process of participants estimating the likelihood of contracting COVID-19 from the activities they attended and making decisions regarding future attendance. This theme raised concerns for the future, feelings of fear and uncertainty for many participants, with some fearful of returning to previous activities:

*I did eventually start going to the supermarket and the chemist. But I haven’t been to any other places. I haven’t been to a shopping centre yet. I hesitate a little to go where there are a lot of people* (Participant 14, 72-year-old female living alone).

Others made the conscious decision not to attend over concerns that social distancing was too challenging, and the risk was too great:

*I go to one night of dancing less a week because of that- too many people there, and I think it’s hard to keep social distance with so many crowded in the room, even though they are allowed to have them, I think it’s too close. As well as being aware of others, a lot of people don’t want to start again. You know they’re old and are a bit frightened of getting into the crowd again* (Participant 7, 73-year-old female living alone).

Participants who had chronic illnesses were particularly fearful of returning to their activities and frustrated at others who were healthy and did not understand the importance of following the rules. This was apparent in views around vaccinations:

*Gardening club has re-started, but I don’t go to it because… If there is someone in there who’s got an active COVID infection, and I get it then my chances of coming out of hospital alive are small…They just don’t get it. They don’t understand it because they are relatively healthy… there are some of us who can’t mix with people who don’t vaccinate* (Participant 13, 67-year-old female living alone).

One participant self-identified as a social person and was aware of her need for community participation. On discussing the possibility of an extended lockdown she decided that she would take a risk:

*I just couldn’t do it. I would take the risk. I would rather take the risk of getting COVID than being that isolated. I could do it for two weeks, quarantine you do it for medical reasons, but to do it just in case? No, I would rather take the risk and let it get me rather than just be so lonely* (Participant 33, 71-year-old female living alone).

Participants reported being fearful of contracting COVID-19 and surprised themselves in their reluctance to return to activities that they thought they would be desperate to get back to:

*The Prime Minister said we strongly suggest people over 70 or anyone who’s got a pre-existing medical condition should stay home. And I thought oh damn, that’s me. So, I rang my neighbourhood watch, my Meals on Wheels coordinator and said “I’m sorry. I have to stop”, and then I sat down and cried for half an hour. I didn’t go back for a while. Eventually, when Meals on Wheels said, “we’re happy to have people back”, I said “no, I think I’ll wait a little bit longer”* (Participant 14, 72-year-old female living alone).

## Reflection and renewal

‘Reflection and renewal’ during the experience of lockdown and social distancing was described, with participants identifying activities they missed:

*I missed my recitals. I noticed it particularly last week because I went to the theatre for the first time in 6 months* (Participant 3, 69-year-old female living alone).

Others noted the things they enjoyed during social distancing, *‘I didn’t feel the obligation to go and interact with people. It was wonderful’* (Participant 1, 75-year-old male living with wife). This reflection for some allowed them to identify changes they would like to make into the future, *‘maybe I don’t want to do the pace that we did before’* (Participant 11, 70-year-old female living alone).

Many participants decided to take up new activities that they had always wanted to do *‘I have signed up to complete 5 kilometres every day for 10 days’* (Participant 22, 74-year-old female living alone) and some made the effort to upskill their use of technology, *‘I’ve learned the - using remote sort of teleconferencing skills, that’s great’* (Participant 33, 71-year-old female living alone).

On reflection, participants were able to identify positives that had come out of social distancing:

*I noticed everybody is cautious about hand sanitising and social distancing. So, I think that is a positive* (Participant 20, 71-year-old female living with friend).

For most participants, reflections turned into renewed appreciation for the activities they were able to return to:

*I felt deprived because of no symphony, no seniors club, no entertainment, no library because I love my volunteering and thank God I am back to that!* (Participant 4, 80-year-old female living alone).

On reflection, several participants mentioned other people they were concerned for, including children’s education and those less healthy and more vulnerable than themselves:

*I’m quite concerned about some of the clients that I’ve been serving… So, I’ve met people, we would do a library delivery. They knew approximately what time we were coming, and we roll up about 11:30 and there are people still in pyjamas. No reason to get up and to get moving. It’s a bit sad isn’t it? So, I am concerned about some of the older folk who I work with that nobody is reaching to them in any way* (Participant 3, 69-year-old female living alone).

## Future planning

In response to the question relating to future planning, ‘*What would you like to see in place to help you keep in touch with people and maintain your physical activity should we experience a second lockdown?’* Overall, participants found it difficult to articulate. Those who successfully redefined their activities during social distancing restrictions reported that they would do the same again:

*We’re like this for the next 12 months, so I’ve adjusted to that, I’m quite happy to be doing things and being very careful, very, very careful* (Participant 22, 74-year-old female living alone).

Others suggested that having means of connection with others was vital, and that technology addressed this need:

*Good technology, good internet, cheaper technology because I spent a lot of money upgrading technology* (Participant 33, 71-year-old female living alone).

## Discussion

This mixed methods study investigated community participation in community dwelling older Australians before, during and after COVID-19 lockdowns. The recruited sample of participants were representative of Australian adults of similar age in health status, levels of obesity and HRQOL [3, 46]. During lockdown, participants took less trips out-of-home, performed more in-home activities, reported fewer social interactions and experienced reduced sleep quality. An overall reduction in MVPA was found during social distancing restrictions. Five themes were identified; ‘*Reframing of meaning, Redefining to maintain activities, Revision of risk, Reflection and renewal* and *Future planning*,’ highlighting the variation in experiences and importance of maintaining community participation.

### Changes in community participation

This study found mixed results for how participants redefined their activities to maintain community participation during restrictions relating to COVID-19. The reduction in the number of trips out-of-home during lockdown was apparent in both quantitative and qualitative data. Conscious attempts to follow the rules were evident for older adults to protect themselves and others they deemed more vulnerable. The number of participants who didn’t leave the house for a whole day increased during lockdown, highlighting that the government’s recommendation to ‘stay home’ were followed. These findings reflect Finnish older adults who reduced the number of destinations they visited during times of social distancing [47].

Commercial locations were visited most frequently across all three time points by older adults, as participants were dependent on these locations to obtain provisions. Likewise, individuals in France experienced consumer attachment, seeking commercial settings for familiarity, authenticity, and security [48]. However, the qualitative data suggestions that access to commercial spaces served a broader purpose of incidental as well as planned connections with others while social distancing. Participants reported informal engagement of waving and saying hello to people as they travelled to commercial locations as having a positive impact upon them. Some participants also reported going to the same locations repeatedly, perhaps to support comfortable familiarity and to replace previous routines. Previous research has highlighted the importance of such casual neighbourhood connections in urban commercial spaces as important to positive aging experiences and well-being, especially for those living alone [49, 50]. This may explain the high number of commercial visits detected or may simply reflect the activities permitted during this time, as Portegijs et al. (2021) reported Finnish older adults only reported locations for exercise [47]. Therefore, differences may be due to variations in restrictions or the number of confirmed COVID-19 cases.

Connections to green space has previously been established as positive for the physical, social, and mental health of older adults [49]. Globally, visits to urban parks increased during the pandemic [51], and were linked with improved, physical, mental and social wellbeing [52] this was reflected in our findings where the number of trips to local walk/greenspaces during lockdown increased. These activities were often redefined to include social interactions which was not the case at baseline, or post lockdown, possibly because participants started returning to their previous activities and routines. However, the evidence suggests that visits to parks and greenspaces combined with social interactions were effective in reducing psychological burden from COVID-19 [53]. This was apparent for our participants as trips to parks and greenspaces allowed them to reframe activities ceased during this time and maintain both their physical and social activities.

A non-significant reduction in minutes of MVPA was found across the time points, which corresponds with 79% of the sample who were less physically active during lockdown. These individuals did not adapt their activities to maintain PA. However, 21% of the sample did adapt to maintain their PA, and on occasion increased it. The vast difference between participants is highlighted by the substantial variation of minutes of MVPA during restrictions, and contrasting personal experiences and approaches during this time, which may explain the insignificant overall results. The reduction of activity in this study is higher than Japanese older adults during COVID-19 restrictions where 47% reported reduced activity levels [54]. This difference may be due to the use of self-reported methods with older Japanese adults, or that at baseline the participants recruited in this study were highly active.

### Impact of restrictions on health-related factors

Overall, HRQOL scores did not change significantly for this sample. Similar findings were reported with US older adults, however, increased feelings of anxiety and less satisfaction with participation were found [9]. In this study, participant HRQOL reduced to match the general population, and independent living and coping scores declined below the normal population averages during and following lockdown [55]. When considered with qualitative data, HRQOL appeared to reduce for individuals who were not successful in reframing or redefining their activities and resulted in a loss of motivation to participate in any activities.

Sleep quality significantly reduced from baseline to lockdown. However, at baseline, numerous participants wore GeneActiv devices during extreme summer temperatures which could have affected these results. The increase in sleep quality between baseline and post lockdown may be due to ‘less worry’ as the situation improved. However, this was not evident in the qualitative data or in other research that found significant reduction in fatigue during times of lockdown [9].

Loneliness levels did not change significantly, however, the sample were not classified as lonely at baseline and the de Jong Gierveld loneliness scale was likely not sensitive enough to detect changes. Despite the validity and reliability of the de Jong Gierveld loneliness scale, it has been acknowledged that the inclusion of positive or negative wording can change the response style [56], which is perhaps why levels of loneliness did not change. However, from the qualitative data it was apparent that some participants struggled with social isolation and feelings of loneliness during social distancing restrictions, yet others were not affected, and some even enjoyed them. Notably, several participants made extra efforts to support social connectedness whilst social distancing, including ringing people every day, connecting with neighbours, and organising socially distanced coffees or walks with friends. Other participants used technology- perhaps for the first time - to keep in contact with family members such as zoom or facetime with grandchildren. Some participants also reported that they were willing to take risks to socialising, viewing social isolation as worse than catching COVID-19. This highlights that older adults are not a homogenous group [57] and perhaps those that enjoyed having more time alone were more introverted and enjoyed the excuse not to socialise.

### Perceptions of community participation

When analysing the results, there were two different approaches to social distancing restrictions. One group accepted that they could not participate in their usual activities and stayed home performing sedentary activities such as crafting and online gaming and the other group adapted their activities to retain them. Those who adapted generally reported more positive experiences and considered others worse off than themselves, which Verhage et al. (2021) suggested allowed older Dutch adults to maintain both self-esteem and self-confidence during COVID-19.

During times of lockdown older adults relied on technology to maintain social connections [9]. Despite the increased use of telephone calls and social media reported in participant diaries, social interactions reduced significantly during this time. Regardless of attempts to maintain social activities, the incidental and informal interactions that came with attending locations, such as recreational facilities, were lost. Concerningly, in a study carried out by van Den Berg (2015) prior to COVID-19, 10% of 213 respondents did not experience any face-to-face social interactions for days, 76% of participants in this study reported days with no social interactions face to face, or otherwise. Which highlights the heightened extent to which social isolation could be experienced by older adults during and following periods of COVID-19 restrictions.

## Limitations

Whilst this study offers a detailed rich picture of community participation and the changes during and following COVID-19, there are limitations that should be acknowledged. The experiences reported mainly represent active older females who lived alone and therefore limits the generalisability of results. The strict lockdown restrictions were only enforced for five weeks in South Australia; therefore, the experiences would perhaps be different in other states where lockdowns were prolonged. Following participant drop out from the baseline sample and large SD of MVPA detected, this research is underpowered to find significant differences in the levels of physical activity for this population. In addition, the baseline levels of MVPA and sleep quality could have been affected by extreme temperatures during data collection meaning participants may have been more active if data collection were carried out over a cooler period (Gough et al., 2021). Despite these limitations this research reports a unique and important perspective of changes to community participation during a period of lockdown.

## Future directions

This research suggests that individuals who adapted their community participation both physically and socially coped better during times of social isolation. It is therefore possible that supportive services aimed at assisting older adults to modify, reframe, and adapt their activities would be useful to reduce loneliness and social isolation. Further research with larger, more diverse samples is required to direct future intervention, as is deeper questioning to determine why older adults changed their behaviours during this time.

## Conclusion

This study suggests that during COVID-19 lockdown, the physical and social activities of community dwelling older adults changed. Older adults reduced their trips out of home yet maintained their trips to commercial locations. Some participants acted to redefine and reframe their activities to maintain them following the closure of recreational facilities, yet others accepted the loss of activities and reported feelings of loneliness and social isolation which they were unable to overcome. This study reports on a sample of older Australian adults who were not lonely at baseline, which raises concerns for those who were socially isolated and/or lonely prior to lockdown and social distancing restrictions. The effects of COVID-19 lockdowns and social distancing on older adults, in lesser health who were frail and vulnerable remains unknown. It is important to identify older adults at increased risk of social isolation. Further research is required to determine whether the experiences of more vulnerable older adults reflect those discussed in this study. The results of this study indicate opportunities to expand public health initiatives to promote healthy ageing in environments such as commercial establishments, where older people are commonly visiting. Furthermore, initiatives that assist older adults to reframe and redefine their activities to adapt to their capacities, to maintain participation and the subsequent health benefits, need to be considered. Fundamental is the codesign of these initiatives with older people to increase both the understanding of their expectations and behaviours to inform future policy, thereby maximising their health and wellbeing through participation.

## Electronic supplementary material

Below is the link to the electronic supplementary material.

Supplementary Material 1

Supplementary Material 2

## Data Availability

The datasets used and analysed during the current study are available from the corresponding author on reasonable request.
