# Supplementary figures and images for: Older adults’ community participation, physical activity, and social interactions during and following COVID-19 restrictions in Australia: a mixed methods approach
Source: BMC Public Health. 2023 Jan 25;23:172. doi: 10.1186/s12889-023-15093-0 (PMC9875767; doi:10.1186/s12889-023-15093-0)

**Additional file 1: Participation diary excerpt**


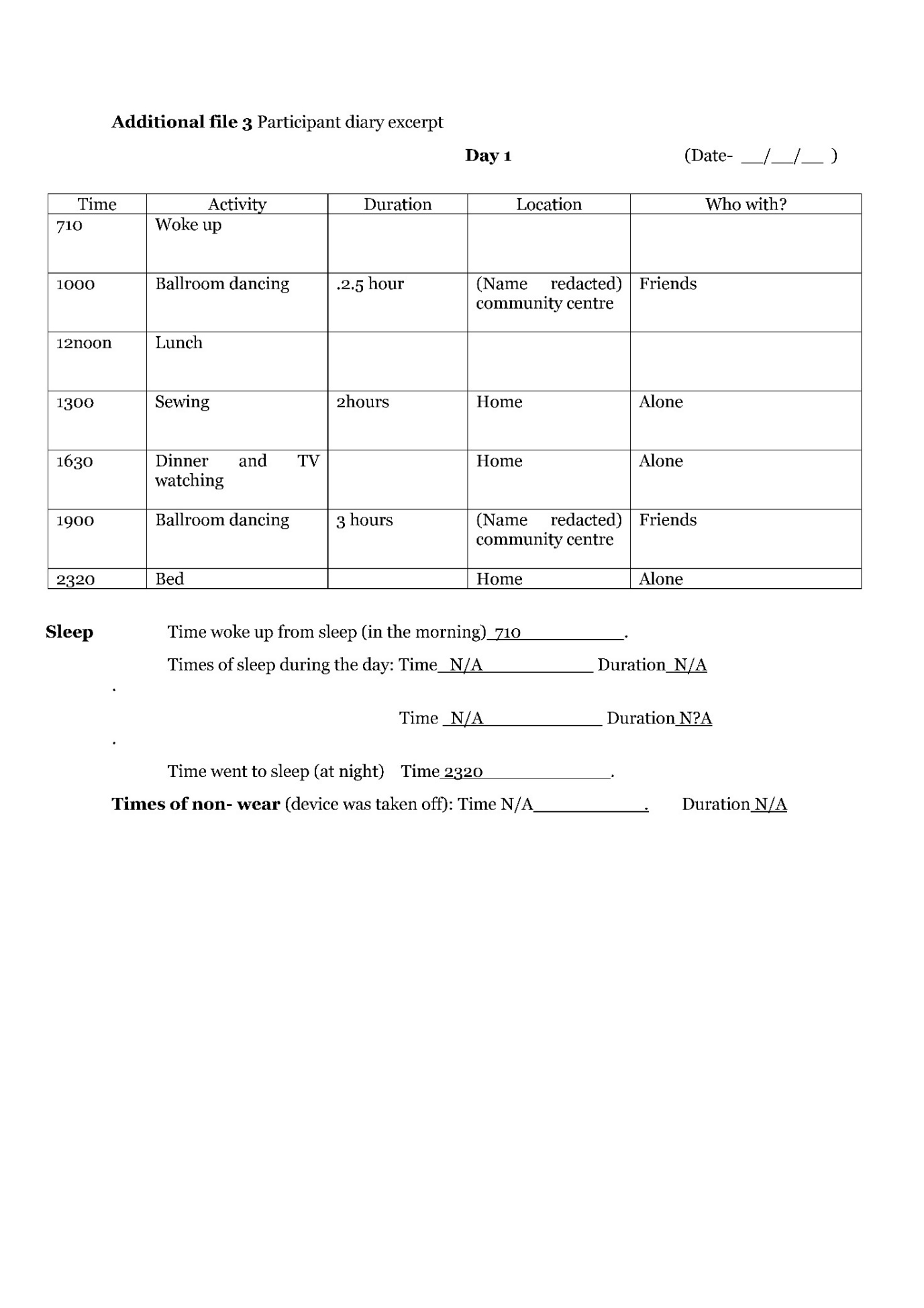

Supplement: Supplementary file 1 — Supplementary Material 1 [file 12889_2023_15093_MOESM1_ESM.docx]

**Additional file 2: Semi structured interview guide**


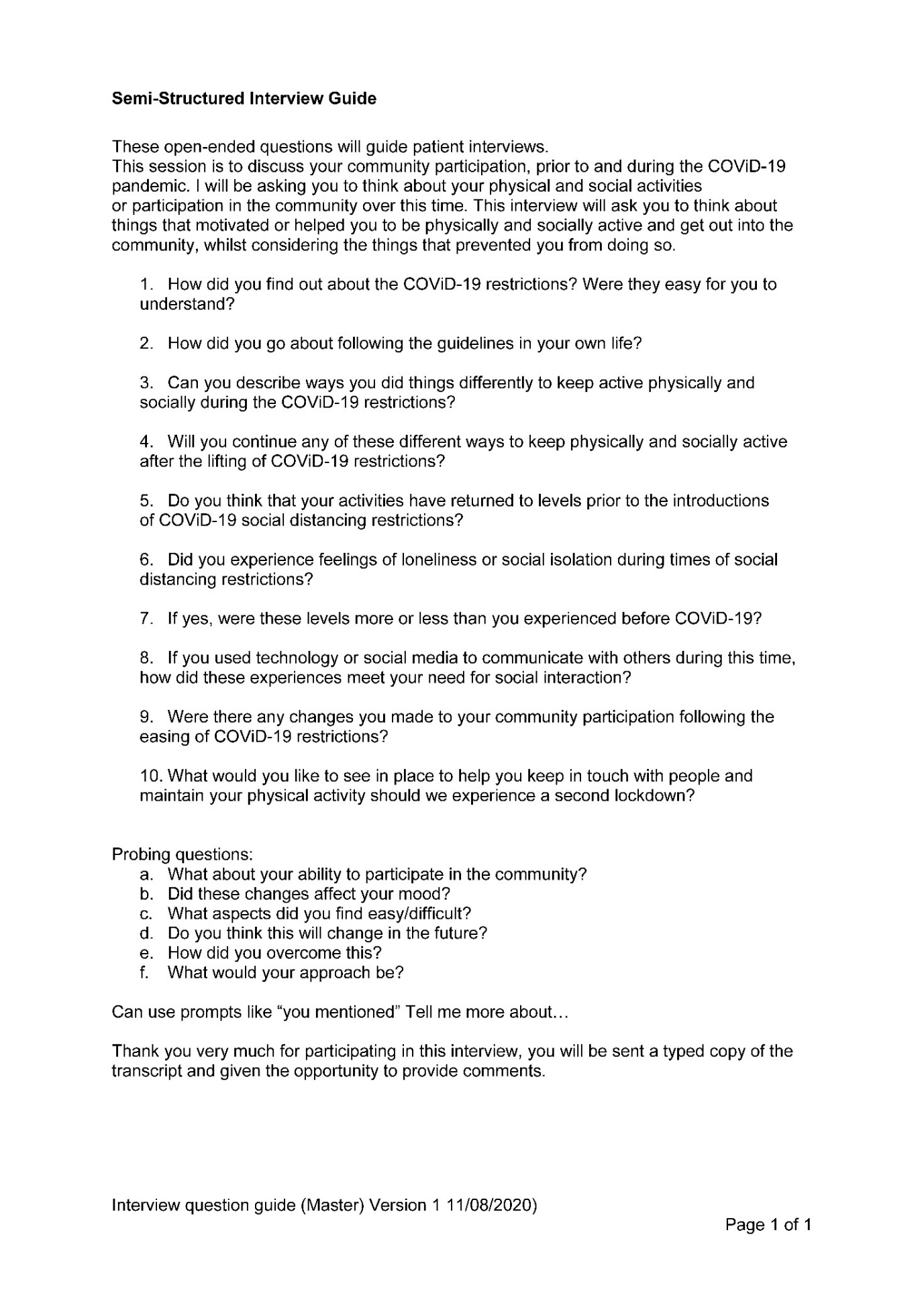

Supplement: Supplementary file 2 — Supplementary Material 2 [file 12889_2023_15093_MOESM2_ESM.docx]
